# Empowerment of disability benefit claimants through an interactive website: design of a randomized controlled trial

**DOI:** 10.1186/1472-6947-9-23

**Published:** 2009-05-10

**Authors:** David Samoocha, David J Bruinvels, Johannes R Anema, Romy Steenbeek, Allard J van der Beek

**Affiliations:** 1 Department of Public and Occupational Health, EMGO Institute, VU University Medical Center, Amsterdam, The Netherlands; 2 Research Center for Insurance Medicine AMC-UWV-VUmc, Amsterdam, The Netherlands; 3 IGER, the Amsterdam Interdisciplinary Centre of Law and Health, Amsterdam, The Netherlands; 4 TNO Quality of Life, Work and Employment, Hoofddorp, The Netherlands

## Abstract

**Background:**

Individuals claiming a disability benefit after long-term sickness absence, have to undergo medical disability assessments. These assessments, often carried out by specialized physicians, can be complicated by wrong expectations or defensive attitudes of disability benefit claimants. It is hypothesized that empowerment of these claimants will enhance the physician-patient relationship by shifting claimants from a passive role to a more active and constructive role during disability assessments. Furthermore, empowerment of claimants may lead to a more realistic expectation and acceptance of the assessment outcome among claimants and may lead to a more accurate assessment by the physician.

**Methods/Design:**

In a two-armed randomized controlled trial (RCT), 230 claimants will be randomized to either the intervention or control group. For the intervention group, an interactive website was designed http://www.wiagesprek.nl using an Intervention Mapping procedure. This website was tested during a pilot study among 51 claimants. The final version of the website consists of five interactive modules, in which claimants will be prepared and empowered step-by-step, prior to their upcoming disability assessment. Other website components are a forum, a personal health record, a personal diary, and information on disability assessment procedures, return to work, and coping with disease and work disability. Subjects from the control group will be directed to a website with commonly available information only.

Approximately two weeks prior to their disability assessment, disability claimants will be recruited through the Dutch Workers Insurance Authority (UWV). Outcomes will be assessed at five occasions: directly after recruitment (baseline), prior to disability assessment, directly after disability assessment as well as 6 and 16 weeks after the assessment. The study's primary outcome is empowerment, measured with the Vrijbaan questionnaire. Secondary outcomes include claimants' satisfaction, perceived justice, coping strategy, and knowledge. A process evaluation will also be conducted.

**Discussion:**

This study evaluates the effectiveness of an interactive website aimed at empowerment of disability claimants. It is hypothesized that by increasing empowerment, the physician-patient relationship may be enhanced and claimants' satisfaction and perceived justice can be improved. Results are expected in 2010.

**Trial registration:**

NTR-1414

## Background

Physician-patient communication is one of the most important tools in health care, and is considered to be an essential aspect of high quality care [1,2]. Research has suggested that effective communication by physicians influences the rate of patient recovery, effective pain control, adherence to treatment regimens, and psychological functioning [2-4]. In order to optimize physician-patient communication, the last decades an extensive amount of studies have been conducted in which interventions were aimed at the physician's role. These interventions consisted mainly of different forms of communication skills training [5,6], and some other methods, such as giving physicians feedback about patient-based surveys [7].

Additionally, research on physician-patient communication has focused on increasing the patients' role in care provision as an important new approach to the improvement of health care [8,9]. The case for patient involvement is based on evidence that patients' active participation during the medical interview is associated with better health outcomes [10,11] and increased involvement improves aspects of medical care [12,13]. Patient-focused interventions to improve physician-patient communication consist of e.g.: information provision [14], helping patients to formulate questions to ask their physicians [15,16], and role-playing exercises that increase attention of behavioural styles [17]. Most of these interventions are based on an empowerment (patient 'activation') approach.

Patient empowerment is defined as *helping the patient to discover and develop the inherent capacity to be responsible for one's own life *[18] and is characterized by a sense of perceived control, self-determination and goal internalization [19,20]. It combines personal efficacy and competence, a sense of mastery and control, and a process of participation to influence decisions and institutions [21]. Previous studies have shown that empowering patients can be effective in improving the physician-patient relationship in primary care [16,22-24]. In the trial described in this article, we introduce patient empowerment into a whole new field of care: insurance medicine.

Through social insurance, workers can claim compensation when they are losing (part of) their income due to disability. To judge these disability benefit claims, disability assessments are carried out by specialized physicians. In these disability assessments, physicians have to make judgments regarding the claimants' medical status and his or her functional capacities concerning vocational rehabilitation [25]. In the Netherlands, these assessments are performed by social insurance physicians who work for the Dutch Workers Insurance Authority (UWV). Worldwide, physicians are involved in similar assessments, even though national practices may vary considerably under social insurance or disability legislation [26].

The way of assessing workers' disability by (insurance) physicians remains a topic of interest and discussion [27-29]. One of the main problems in adequately evaluating disability claims lies in the physician-claimant interaction [30]. Compared to the 'normal' physician-patient relationship, where a physician has a caring and therapeutic role, this specific physician-claimant relationship is complicated by the fact that insurance physicians have to engage in different kinds of roles (sometimes roles they are not used to): i.e. being a source of information; advocate and counselor; and adjudicator and certifier [31]. Consequently, many physicians report feeling uncomfortable with performing disability assessments [32].

On the claimant side, it often has been stated that a passive and defensive attitude during disability assessments causes strain in the physician-claimant relation. This claimant behaviour can be attributed to three factors.

First, social security arrangements often stimulate passive and defensive behaviour. The fact that claimants have to prove that they are ill (in order to receive a disability pension) causes problems in the assessment of disability and discourages claimants to return to work [33,34].

Second, it has been stated that disability claimants are often characterized as being passive because of the perception that their capacity is limited and their odds of returning to work is low [35]. This behavioural characteristic frequently has a greater influence on the duration of recovery than health-related factors such as the severity of the disability [36]. In the disability assessment, this often leads to discrepancies in views between the physician and the claimant on the claimants functional capacity.

Third, complicated and not fully transparent disability assessment procedures and social security arrangements cause a lack of claimants' knowledge and understanding about this topic and results in claimants frequently having wrong expectations of disability assessment outcomes. From this perspective, patient empowerment is expected to influence the process of disability assessment in a beneficial way.

As a consequence of the complicated relationship between physicians and claimants, many claimants experience disability assessments as injustice [30,37] and reports indicate that patient satisfaction with insurance physicians is lower than, for example, occupational physicians [38].

In an attempt to improve the insurance physician-claimant interaction, the present study aims at empowering claimants prior to their disability assessment. Patient (or claimant) empowerment is expected to strengthen the sense of control among the claimant, so that more directed information concerning his or her disability can be shared with the physician. This, in turn, can lead to a better relationship between physicians and claimants. The classical physician-patient relationship, where the interaction is dominated by the insurance physician, can make room for a more collaborative relationship: the so-called "physician-patient partnership" [39]. Additionally, empowerment of claimants may also lead to a shift in claimants' perception of their limitations, and in a more positive attitude towards return to work.

Because the Internet has a profound impact on health care and has the potential to empower and educate patients effectively, to support decision making, and to enhance the interaction between health consumers and professionals [40,41], the purpose of this study is to empower patients through an interactive website intervention. Next to existing evidence on the effectiveness of eHealth interventions [42], the Internet has the possibility to deliver information to a large audience easily and at a low cost. This will improve the chances of implementation of this pragmatic intervention.

In this article, we describe the design of a randomized controlled trial (RCT) of which the aim is to evaluate the effectiveness of an interactive website designed to increase empowerment of disability claimants. It is hypothesized that this website intervention will:

1. Increase empowerment among disability claimants.

2. Enhance the physician-patient relationship, so that both are more satisfied with the interaction during disability assessment.

## Methods/Design

The project *Empowerment *is a randomized controlled trial with four months of follow-up. Randomization will take place to two groups: an intervention group using an interactive website and a control group using a website with commonly available information only. The recruitment and data collection for this study started in January 2009.

The study design and procedures were approved by the Medical Ethics Committee of the VU University Medical Center (under number 08/194).

### Study population

Participants are claimants for a disability pension according to the Dutch *Work and Income Act (WIA)*, which can be claimed after being sick-listed for 104 weeks. All disability claimants will be recruited in three different offices of the Dutch Workers Insurance Authority, UWV (Leiden, Den Haag, Rotterdam). UWV is responsible for evaluating disability claims in the Netherlands. Claimants will receive an invitational letter for the disability assessment including a study information brochure. The latter will direct claimants to an online application form. This application form includes questions concerning the study's in- and exclusion criteria and an informed consent. Recruitment will take place over a 8-month period (January 2009 – August 2009).

All insurance physicians from the participating UWV offices, and responsible for disability assessments concerning the Dutch *Work and Income Act (WIA)*, were asked to participate in the study.

### Inclusion & exclusion criteria

For claimants, inclusion criteria of the study are:

1) Adequate knowledge of the Dutch language,

2) Having an email address.

Claimants will be excluded if they had had any disability claim assessment in the past.

### Sample size

A power analysis has been carried out for the main outcome measure, i.e. empowerment. Empowerment will be measured using the "VrijBaan" questionnaire [43]. This questionnaire consists of six subscales. Based on previous findings the expected standard deviation (SD) for each of these scales is 0.70 [43]. Power calculations indicate that, to detect a 10% difference in empowerment, 86 subjects are necessary in each study group (assuming that power = 0.90 and α = 0.05). Taking into account a drop out rate of 25%, a sample size of approximately 230 claimants will be required.

### Randomization

Randomization will be conducted at the individual claimant level. After baseline measurements, disability claimants will be randomized in either the intervention or control group. Randomization to these two groups will be done by block randomization. To prevent unequal groups, 3 blocks will be created (3 UWV offices). A computerized random number generator will draw up an allocation schedule for each block. In consequence of the nature of the intervention (interactive website vs. a 'sham' website with commonly available information), claimants will be blinded for study design. Insurance physicians will be aware of the study's design, but will not be informed about the allocation of disability claimants.

### Development of the intervention

To determine the specific content of the website, the process of Intervention Mapping was used as a supportive tool [44]. The following steps were taken:

#### Step 1: needs assessment

In May 2007 we started with a needs assessment. In this step we performed semi-structured interviews with 8 insurance physicians and two labour experts. During these interviews we asked these stakeholders what problems they encounter with claimants during disability assessments and what their needs were in their interaction with claimants.

Furthermore, we conducted a survey that was returned by 41 disability claimants who had had a disability assessment in the preceding month. In this survey we asked claimants how they had prepared for their disability assessment, what problems they had experienced and what suggestions or needs they had for the intervention.

#### Step 2: defining objectives

On basis of the needs assessment, we defined the intervention and learning objectives. These were to:

- Increase claimants' knowledge about social security arrangements and disability assessment procedures.

- Provide claimants with realistic expectations on disability assessment outcomes.

- Increase claimants' skills and self-efficacy to actively participate during their disability assessment.

- Give claimants the opportunity to interact with other claimants in order to increase social support.

- Increase claimants self-awareness on their functional possibilities and limitations, so that they are able to communicate this better with their physician.

- Increase 'general' empowerment of claimants by, for example, giving information and advice on how to cope with disease and work disability.

#### Step 3: systematic review

Next, we performed a systematic review in which studies that were aimed at increasing empowerment through online tools were retrieved. Interventions that were found to be successful in increasing patient empowerment were well studied in order to obtain insight into the effective elements. For example, we used synchronized audio and text fragments supported by video (so-called 'modules'), comparable to a study by Warmerdam et al. [45].

In step 3 we also contacted patient organisations experienced in issues around disability assessments and asked them for methods suitable for the intervention. Existing brochures and websites that contain information on how to prepare for a disability assessment were consulted for inspiration as well.

#### Step 4: focus groups

Based on the previous steps, a first concept of the intervention was developed. With this concept in mind, we held two focus groups in which we discussed the intervention with a group of six insurance physicians and a group of five claimants and representatives of patient organizations respectively. With feedback and input from the focus groups the concept was further developed. Some ideas for the intervention were omitted when support was lacking.

#### Step 5: website development

In September 2008 the beta version of the website http://www.wiagesprek.nl was launched online. In the following four months the site was beta tested by professionals and a representative of a patient organization. Additionally, we conducted a pilot study in this period among 51 claimants. On basis of obtained feedback from the professionals as well as the results of an evaluation form filled out by 30 of these 51 claimants we adapted the website into a final version.

### Description of the intervention

The final web-based intervention http://www.wiagesprek.nl will consist of several components:

First, the website will contain general information and features concerning absenteeism from work, including:

- Social security law arrangements, with simple explanations of the WIA and its procedures

- Disability assessment procedures, including detailed advice on how to prepare for a disability assessment

- Return to work information (e.g. information on how to approach rehabilitation offices)

- Information about patient organizations

- Personal experiences of people who underwent WIA procedures

- Coping with disease and work disability

- Links to other related websites.

Second, the site will contain an extensive forum that gives participants the ability to interact with other claimants who are in the same situation. This interaction could be about, for example, issues concerning their disability assessment or coping with disease and work disability. Information on the forum will be updated by a moderator, who will also answer questions on the discussion forum posted by participants.

Third, participants will be asked to complete five interactive lessons or 'modules'. Each module will prepare participants step-by-step for their consultation visit with the insurance physician of UWV. Participants will be able to finish the modules in their own pace in a period of approximately two weeks prior to their disability assessment.

In *module 1 *(~20 min), Dutch legislation procedures will be explained to subjects in order to increase subjects' knowledge about WIA procedures and the exact content of disability assessment. An interactive quiz will test subjects' knowledge at the end of the module.

*Module 2 *(~20 min) focuses on the consultation visit with the insurance physician of UWV. Subjects will be asked to fill out their medical record and keep up an online diary that will prepare them for the actual disability assessment.

In *module 3 *(~15 min), videos of patient-physician interaction will be shown to subjects in order to teach them how to actively participate during their consultation visit with the insurance physician.

In *module 4 *(~15 min), expectations of subjects' disability claim outcomes will be discussed. Also, an interactive tool (the "WIA meter") will help subjects to increase their self-awareness and will test their motivation to return to work.

*Module 5 *(~5 min) will summarize all previous modules and will discuss the 6 most important tips concerning preparation for the upcoming disability assessment.

Throughout the modules subjects will be asked to finish short assignments in order to increase motivation and self-awareness about their disease and situation.

See [Additional file 1] for screenshots of the website intervention.

### Study groups

All participants will be able to access the homepage of the website http://www.wiagesprek.nl. From this homepage, participants from the intervention group can enter the Internet intervention with a username and password. These will be sent to participants after they have filled out an online application form. Comparably, participants from the control group will be directed to a website with commonly available information only. This control group website will only contain brief information about disability assessment procedures, which can normally be found in standard UWV brochures.

### Study procedures

**Figure 1:**
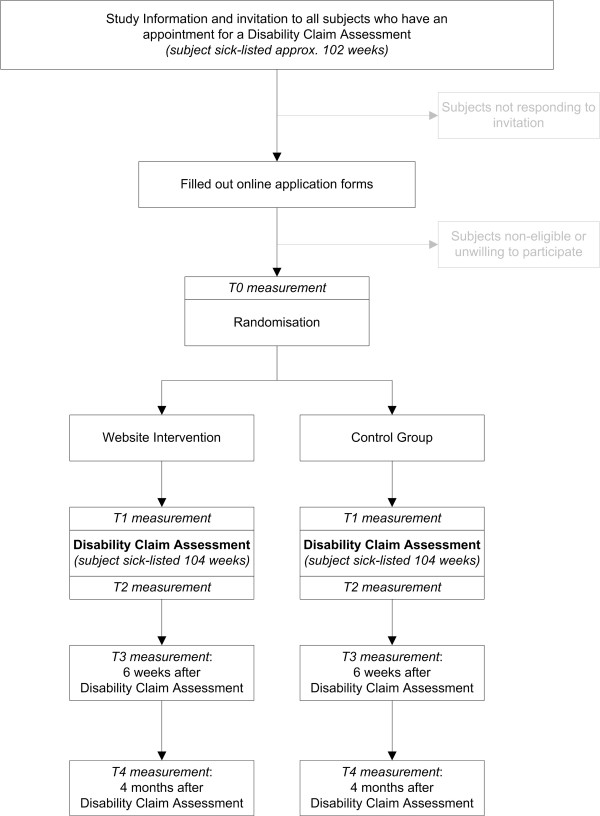
**Overview of study procedures**.

The study design is presented in Figure 1.

In the Dutch social security system, workers that are long term sick-listed can apply for a disability benefit at approximately 91 weeks after the start of their sick leave. Around the 102^nd ^week of being sick-listed, these workers will receive an invitation from UWV to visit an insurance physician,. Together with this standard invitation from UWV, this study will send along an invitational letter and the study's information brochure. In this brochure, instructions are given on how to participate in the study by filling out an online application form. This online form includes questions concerning the study's in- and exclusion criteria and an informed consent. Subjects who meet all the criteria and are willing to participate in the research will be directed to the baseline questionnaire (T_0_). When subjects finish filling out the baseline questionnaire, they will be randomized in either the intervention group or control group. Disability claimants who are randomized into the intervention group, will receive an email with a username and password that gives them full access to the website intervention. Participants who are randomized into the control group receive an email that directs them to a website with commonly available information only. Before each follow-up measurement, subjects from both groups will receive emails in which they are asked to fill out the online questionnaires. Reminders will be sent to increase compliance.

Insurance physicians receive a newsletter with information about the ongoing of the project. Directly after a disability assessment with a claimant participating in the study, insurance physicians will be asked to fill out a short questionnaire, which they will receive by email.

#### Outcomes

Measurements will take place on five different occasions:

- T_0_: Baseline measurement. Approximately 2 weeks before the disability assessment.

Questionnaire filled out by claimants.

- T_1_: Two days before the disability assessment.

Questionnaire filled out by claimants.

- T_2_: Directly after finishing the disability assessment.

Questionnaires filled out by claimants and insurance physicians.

- T_3_: Six weeks after the disability assessment. At this point, claimants will be informed on the decision about their disability pension.

Questionnaire filled out by claimants.

- T_4_: Four months after the disability assessment, a follow-up measurement will take place.

Questionnaire filled out by claimants.

All outcomes will be measured through online questionnaires [see Additional file 2]:

### Primary outcome

#### Empowerment

The main outcome measure will be empowerment. Because of the absence of a generally accepted measurement scale for this variable, the so-called "VrijBaan questionnaire" was developed: an instrument that has been designed to measure empowerment among people with a work disability [43]. The VrijBaan questionnaire consists of 60 items divided over six subscales (Competence:13 items, Self-determination:11 items, Meaning:9 items, Impact:8 items, Positive Identity:10 items, Group Orientation:9 items). Each subscale contains components of previously validated instruments that measure specific components of empowerment (such as the General Self-efficacy scale [46] or the Pearlin Mastery Scale [47]).

Internal consistency of the VrijBaan questionnaire was found to be good. After testing the questionnaire in a representative population (n = 385) all subscales showed Cronbach's alphas higher than 0.80 [43].

Due to the length of the questionnaire we choose to only include the subscales Impact and Competence in the T_1 _measurement. Furthermore, in order to make international comparisons with other empowerment-related studies we added five questions of the General Self-efficacy scale and two questions of the Pearlin Mastery Scale that were not included in the Vrijbaan questionnaire. The constructs measured with these scales (self-efficacy and mastery respectively) are often associated with empowerment.

Finally, we added two adapted questions from the Pearlin Mastery Scale in order to measure context specific empowerment.

### Secondary outcomes

#### Claimants satisfaction

The satisfaction of claimants with their insurance physicians will be measured with the AStri questionnaire [48]. This questionnaire, which is specially designed to measure patient satisfaction in the field of insurance medicine, contains 29 items divided over six subscales, each representing a different component of patient-insurance physician interaction (Listening, Empathizing, Correctness, Clearness, Rigorousness, and Professionalism). Testing of this questionnaire resulted in a good internal consistency (all subscales had a Cronbach's alpha greater than 0.78) [48].

#### Physicians satisfaction

Insurance physicians satisfaction with the disability assessment and claimants attitude will be assessed with a questionnaire specially designed for this study. In this 10-item questionnaire physicians can answer questions on a 5-point Likert scale ranging from *I totally disagree *to *I totally agree*. In addition, physicians will be asked how actively claimants were participating during the assessment and how much time they spent on the assessment.

#### Claimants perceived justice

To measure claimants feelings of justice with the final verdict on their disability pension, a Dutch translation of Moorman's [49] justice questionnaire will be used [50]. This questionnaire consists of 30 items measuring three dimensions of justice perceptions: distributive justice (the perception of fairness of the outcomes a claimant receives; 7 items), procedural justice (the perception of fairness of the procedures used to determine these outcomes, 12 items), and interactional justice (the perception of fairness of contact with the organization that determines the outcomes, 11 items). Each item can be scored on a 7-point scale ranging from "*I totally do not agree*" (1 point) to "*I totally agree*" (7 points). Average scores are calculated for each separate dimension. In the present study we will only use the subscales distributive justice and procedural justice. Cronbach's alpha for these dimension of the questionnaire was proven to be high (distributive justice: α = 0.91, procedural justice: α = 0.82) [51].

#### Subjective knowledge

With a 10-point Visual Analogue Scale (VAS), we will measure claimants subjective knowledge about social security law arrangements and disability assessment procedures. At T_0 _we will ask: "How much do you know about social security law arrangements and disability assessment procedures?" (1 = I know nothing, 10 = I know everything). At T_1 _we will ask: "To what extent did the intervention increase your knowledge about social security law arrangements and disability assessment procedures?" (1 = my knowledge did not increase, 10 = I gained maximum knowledge).

#### Claimants expectation

Questions concerning expectations of claimants on the outcomes of their disability assessment will be asked at T_0_, T_1 _en T_2_. Subjects will be asked whether they expect to receive a disability benefit after the disability assessment (yes/partly/no) and the reason for this belief.

#### Coping strategy

To evaluate if the intervention also might lead to a different coping strategy within claimants, we will measure coping strategy with the Dutch adaptation of the Ways of Coping Questionnaire (WCQ, [52]). This questionnaire is based on Lazarus' Theory of Stress and Coping [53], which states that coping is situation-specific rather than a trait or disposition. Three dimensions of the WCQ are included: Problem Solving (8 items), Seeking Social Support (6 items) and Avoidance (7 items). Questions from these scales are adapted to the context of the disability assessment.

#### Perceived Work Ability

Perceived work ability will be assessed with the first item from the Work Ability Index (WAI) [54]. This question asks subjects to rate their current work ability compared to their lifetime best on a 10-point scale, ranging from *completely unable to work *(score '0') to *work ability at its best *(score '10').

### Other variables

#### Socio-demographic

At baseline, socio-demographic data (gender, age, level of education, current work status, working hours per week, nationality, Internet use and disability type) will be collected.

#### Claimants preparation

Claimants from the intervention group and control group will be asked about their preparation for their disability assessment at the T_1 _measurement. Questions such as "How much time did you spend on gathering information about disability assessment procedures" or "Which websites did you visit to obtain information about the WIA benefit" will be asked.

Additionally, use of the website intervention (e.g. amount of mouse-clicks on the website, total login time, use of particular components) will be collected up to four months after the disability assessment.

#### Return to work (RTW)

With data from UWV we will determine claimants working status four months after disability assessment. Return to work data will be categorized into full, partial or no return to work.

After finishing the study's inclusion period, we will obtain data from UWV on claimants' official objections and appeal.

### Process evaluation

Among the participants of the intervention group, experiences with the use of the intervention will be evaluated. This evaluation will be assessed with a brief questionnaire at T_3_. This questionnaire contains both quantitative (e.g. a generic grade for the website) and qualitative questions (e.g. "Do you have any suggestions to improve the website?"). Reasons for complying or not complying will be asked in this questionnaire as well, in order to obtain insight into the potential success of implementation.

Furthermore, the website http://www.wiagesprek.nl will automatically collect data on website usage, so that we can determine compliance and which components are most frequently used. All actions on the website of each claimant will be tracked and stored in a database.

After finishing the research, we will interview five claimants and five insurance physicians, who assessed disability of study participants, on their experiences with the intervention.

For an overview of measurements, see table 1.

**Table 1:** Schedule of measurements

Outcome measure	T_0_: 2 weeks prior to disability assessment	T_1_: Prior to disability assessment	T_2_: After disability assessment	T_3_: 6 weeks after disability assessment	T_4_: 4 months after disability assessment
Empowerment*	X	X			X

*Claimants satisfaction*			X		

*Perceived Justice*				X	

*Subjective knowledge*	X	X			

*Claimants expectations*	X	X	X		

*Coping Strategy*	X	X			

*Perceived Work Ability*	X	X			X

*Claimants preparation*		X			

*Return to Work*					X

*Process Evaluation*				X	

*Physicians satisfaction*			X		

* The following subscales from the VrijBaan empowerment questionnaire are included in the questionnaire:

T_0_: all subscales

T_1_: subscale Competence and Impact

T_4_: all subscales

NB: The Pearlin Mastery Scale and the General Self-efficacy scale can be distracted from the T_0_, T_1 _and T_4 _measurement

### Statistical analysis

Baseline values will be analyzed for differences between the two groups, by one-way-analysis of variance for numerical data and chi-square for categorical data. The primary analysis will be a comparison of the change in empowerment between the intervention and control group following the "intention-to-treat" principle. On data from questionnaires filled out on more than one occasion (e.g. empowerment, coping strategy, knowledge) we will use a repeated measurement analysis to test if both research groups diverged from baseline to follow-up. With data that will be measured on one occasion (e.g. claimant satisfaction, insurance physicians satisfaction and perceived justice) we will use t-tests to detect possible differences between the two research groups. Additionally, a per-protocol analysis will be performed.

## Discussion

In many countries, workers with long-term sickness absence can claim a disability benefit. For these patients, the assessment of their disability claim is an important life event and outcomes of these assessments determine important aspects in a patients' life, such as financial certainty and future work status. Although stakes are high for the involved claimant, the general idea is that these patients are passive, not only during the consultation visit with the physician who assesses their disability, but also when searching for possibilities in order to return to work. This attitude will not only complicate the physician-patient relationship, but also aggravate claimants return to work perspectives. Therefore, in the present study we developed the interactive website http://www.wiagesprek.nl.

We applied the IM protocol in the development of this intervention [44]. This protocol proved to be a useful tool for tertiary prevention in occupational health, because it combines a theory-, evidence- and practise-based approach [55]. With the help of the IM protocol important stakeholders, such as claimants and insurance physicians, are involved in the development of the website and definition of learning objectives. For this reason it is hypothesized that the applicability and compliance of these stakeholders will be enhanced.

The aim of the interactive website will be to prepare and empower patients prior to their disability assessment. First, based on existing evidence in other fields of research [56], we hypothesize that empowerment of disability claimants will lead to improvement of the physician-patient communication, operationalized by the outcomes patient satisfaction and physician satisfaction. Improved communication will lead to a better information provision by the claimant which, at its turn, can lead to a more accurate assessment by the physician [57]. Second, empowerment may lead to more knowledge about social security arrangements and disability assessment procedures that will influence claimants' expectations on the outcomes of their disability assessment. Consequently, realistic expectations will have an effect on the perceived justice that claimants experience during the process of determination of their disability benefit. Although not the study's primary objective, we furthermore hypothesize that empowerment of disability claimants may lead to an increased return to work status.

To our knowledge, this is the first study in the field of disability management that focuses on an eHealth approach aiming at empowerment of patients, which contributes to a more active participation of claimants in the disability assessment. One of the main strengths of this pragmatic trial is that it uses an innovative method that is easily accessible by a relatively high number of claimants in the Netherlands at a low cost. Once proven effective, this will make chances of a nationwide implementation high.

We choose the Internet as the method to deliver the intervention, based on evidence regarding the effectiveness of online interventions on behavioural change outcomes in general [42], and more specifically, on patient empowerment [58,59]. Also, literature suggests that there are many advantages of using the Internet to deliver health care interventions. These advantages include the unique characteristics of Internet technology (e.g. the use of video transmission techniques), its cost-effectiveness, the possibility to reach isolated or stigmatized groups, the timeliness and easy access of information or advice, and the flexibility of user control of the intervention [60]. However, there are also disadvantages of using the Internet to deliver health care interventions. A disadvantage of web-based interventions which also may play a role in the present study, is that compliance in web-based interventions is sometimes low [42]. However, by adapting the intervention to the specific needs of the stakeholders (as was conducted by using the IM protocol) we expect to reduce the risk of low compliance in this intervention.

From a research perspective, the results and process evaluation of the current study will give insight in methods to enhance patient-physician communication as well as insight in the effectiveness of online tools to increase patients' participation in the patient-physician encounter.

There are also some limitations. The most important limitation is that this study may possibly suffer from selection bias. Results from our pilot study showed that probably a large proportion of the claimants we will approach, will not participate in the study. If characteristics from these non-participants differ substantially from participants, the validity and applicability of the study results will be threatened [61]. In a trial aiming at empowerment this especially may play an important role, because of the expected higher response among more empowered claimants than among those who are less empowered. We therefore hope that a proper non-response analysis will point out that the results are not restricted to a selected population.

Results of the *Empowerment *study will be available in late 2010. If the results are positive, the website http://www.wiagesprek.nl may be implemented nationwide after possible adjustments on basis of the study's conducted process evaluation.

## Competing interests

The authors declare that they have no competing interests.

## Authors' contributions

DB wrote the initial study protocol. DS and DB designed the intervention protocol and the website. DS wrote the manuscript, which was commented on by DB, RS, HA and AB. All authors have read and approved the final version of the manuscript.

## Pre-publication history

The pre-publication history for this paper can be accessed here:

http://www.biomedcentral.com/1472-6947/9/23/prepub

## Supplementary Material

Additional file 1 Screenshots http://www.wiagesprek.nl. Some screenshots of the intervention http://www.wiagesprek.nl. Click here for file

Additional file 2 Questionnaires. Detailed description of the questionnaires used in this study. Click here for file
