# Supplementary material for: Empowerment of disability benefit claimants through an interactive website: design of a randomized controlled trial
Source: BMC Med Inform Decis Mak. 2009 May 10;9:23. doi: 10.1186/1472-6947-9-23 (PMC2689177; doi:10.1186/1472-6947-9-23)
Supplement: Additional file 1 — Screenshots http://www.wiagesprek.nl. Some screenshots of the intervention http://www.wiagesprek.nl. [file 1472-6947-9-23-S1.pdf]

eMGO+
Institute for Health and Care Research

English

Welkom op WIAgesprek.nl

WIAgesprek.nl is dè site om u voor te bereiden op de WIA-beoordeling bij het UWV. WIAgesprek is onderdeel van het onderzoeksproject *Empowerment*, uitgevoerd door het VU medisch centrum. Klik hier voor meer informatie over het onderzoek.

Bezoekt u deze site voor het eerst? Klik dan voor het aanvragen van een wachtwoord op **aanmelden**. Met dit wachtwoord kunt u vervolgens hieronder inloggen.

Login

gebruikersnaam

wachtwoord

OK

Wachtwoord vergeten?

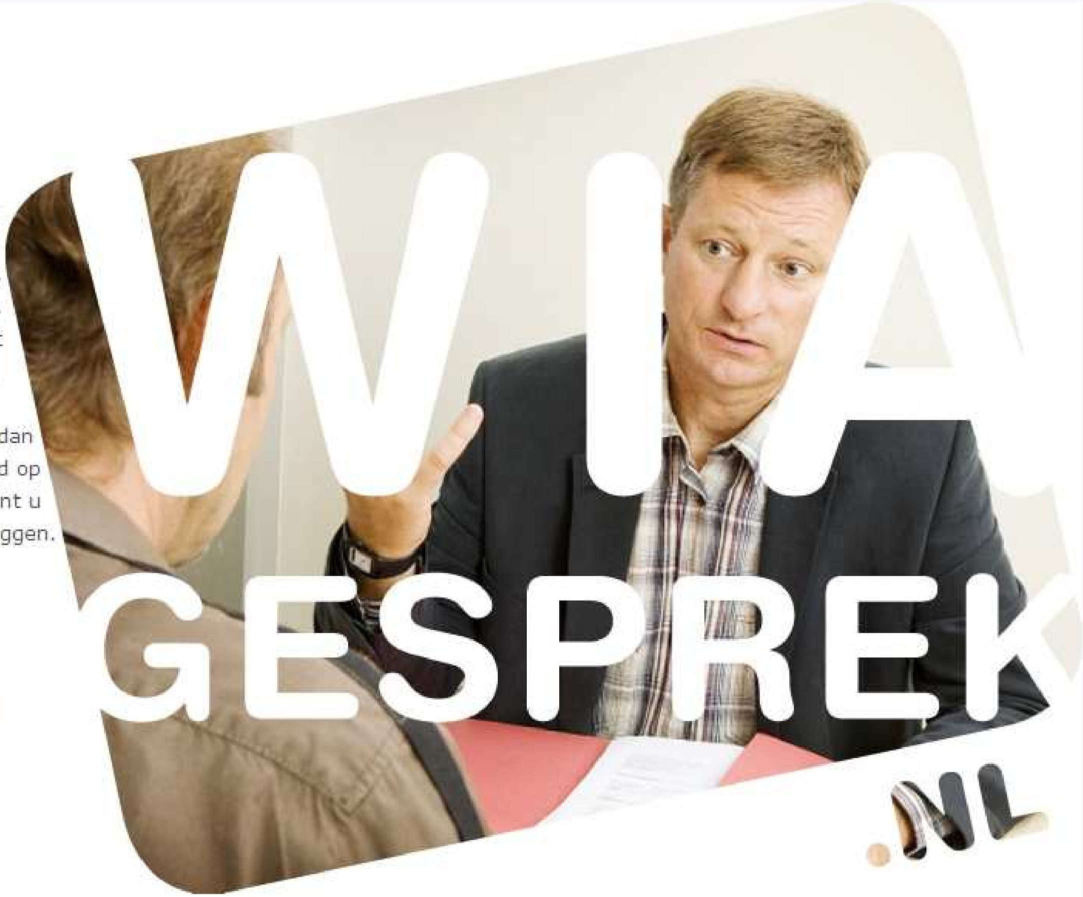

eMGO+ Institute for Health and Care Research

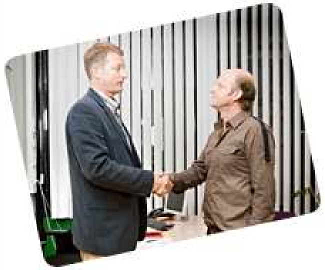

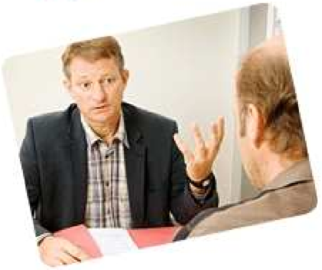

A A A

Het gesprek met de verzekeringsarts

Het gesprek met de verzekeringsarts bij het UWV duurt ongeveer 30 tot 60 minuten. In deze tijd bekijkt de arts uw gezondheid, klachten en vooral wat u nog kunt doen ondanks uw klachten. Dit doet de arts door vragen aan u te stellen aan de hand van uw medisch dossier. Soms doet de arts ook een lichamelijk onderzoek.

De verzekeringsarts
De verzekeringsarts van het UWV is geen gewone dokter. Hij of zij schrijft geen behandeling of medicijnen voor. De verzekeringsarts heeft als taak te kijken wat u wel en niet kan met betrekking tot werk. Verwacht dus niet dat de verzekeringsarts uitgebreid stilstaat bij uw klachten. Hij of zij zal meer tijd besteden aan wat u nog wèl kunt ondanks uw ziekte.

Welke vragen kunt u verwachten?
De verzekeringsarts stelt u een aantal vragen. Zo bepaalt hij of zij wat u wel en niet kunt. De belangrijkste vragen zijn:
- Welke klachten heeft u?
- Hoe ontwikkelen uw klachten zich? Heeft u nu bijvoorbeeld meer of minder klachten dan een paar maanden geleden?
- Wat kunt u nog wel doen ondanks de klachten die u heeft? En wat niet?
- Welke behandeling(en) heeft u (gehad)?
- Wat zijn de gevolgen van uw klachten voor uw werk?
- Kunt u beschrijven wat u zoal doet op een dag?

Bij elke vraag zal de arts doorvragen. Zo probeert hij of zij meer duidelijkheid te krijgen over uw situatie.

Waar houdt de verzekeringsarts rekening mee?
Bij de beoordeling kijkt de verzekeringsarts vooral naar uw mogelijkheden (wat u nog wel kan). Het gaat dus niet zozeer om wat voor ziekte u heeft. Maar meer om wat deze ziekte voor ù betekent. En welke beperkingen u heeft door uw ziekte.

De Functionele Mogelijkheden Lijst (FML)
Aan het eind van het gesprek vult de arts de Functionele Mogelijkheden Lijst (FML) in. Hierop staan uw beperkingen, volgens [illegible]

WIA GESPREK .NL

AANTAL DEELNEMERS WIAGESPREK.NL: 178
AANTAL DEELNEMERS ONLINE: 1

HOME | COLOFON | PRIVACY | HELP | DISCLAIMER | UITLOGGEN

DE WIA | DE WIA BEOORDELING | NA DE BEOORDELING | ZELFREGIE | LINKS

OMGAAN MET ZIEKTE | HULP VRAGEN | VAARDIGHEDEN

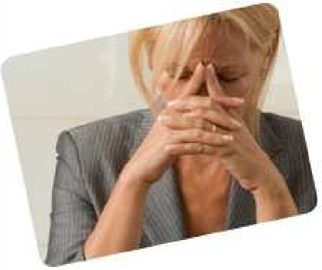

Omgaan met ziekte

Het is lastig om op een goede manier om te gaan met een ziekte. Het aanvaarden van uw ziekte is erg belangrijk. Nog belangrijker is controle hebben over de ziekte waar u mee te maken heeft.

Tips

TIP

Probeer meer te weten te komen over de ziekte die u heeft. Word bijvoorbeeld lid van de patiëntenvereniging die gespecialiseerd is in uw aandoening. Een overzicht van alle patiëntenverenigingen vind u hier. Via deze verenigingen krijgt u contact met mensen met dezelfde ziekte. Of kijk eens op het WIA-forum van deze site. Er worden vaak ook voorlichtingsbijeenkomsten gehouden over uw ziekte. Het is belangrijk deze bijeenkomsten te bezoeken.

TIP

A A A

Lees veel over ervaringen van andere mensen die dezelfde ziekte hebben. Of lees verhalen van professionals die veel weten over een bepaalde ziekte. U kunt via internet ook een van de volgende boekjes bestellen:

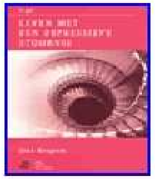

Leven met een depressieve stoornis

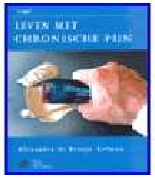

Leven met chronische pijn

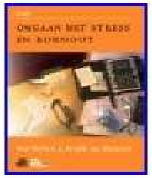

Omgaan met stress en burnout

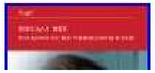

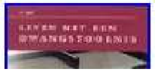

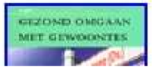

MODULES / OPDRACHTEN

FORUM

MIJN WIA

AANTAL DEELNEMERS WIAGESPREK.NL: 178
AANTAL DEELNEMERS ONLINE: 1

eMGO+ Institute for Health and Care Research

IK WIL GAAN — IK WIL BLIJVEN

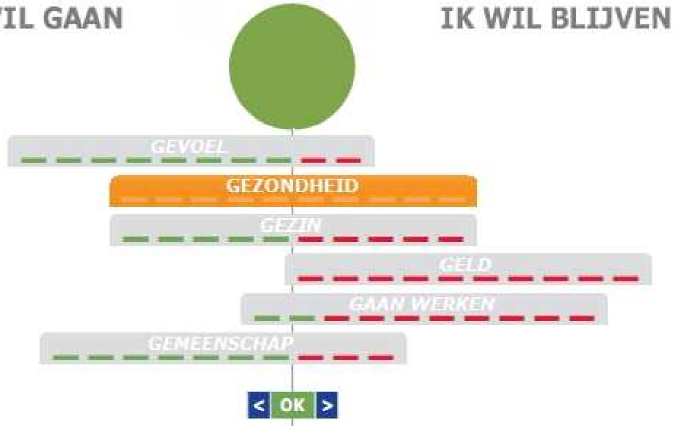

GEZONDHEID

- [ ] Ik ben bijna beter.
- [ ] Het gaat beter met me dan voorheen, ik kan best in deeltijd werken.
- [ ] Werken zou goed zijn voor mijn gezondheid.
- [ ] Ik wil mijn eigen leven niet langer laten bepalen door mijn ziekte.
- [ ] Door te (blijven) werken, zou ik sneller beter worden.

- [x] Ik ben te ziek om (meer) te gaan werken.
- [x] Ik wil mijn klachten niet verergeren.
- [x] Ik heb meer tijd nodig voor herstel.
- [x] De dokter zegt dat ik te ziek ben om te werken.
- [ ] Als ik meer zou werken, zou ik zieker worden.
- [ ] Zolang ik niet werk, kan ik goed met mijn beperkingen omgaan.

WIA GESPREK .NL

AANTAL DEELNEMERS WIAGESPREK.NL: **178**
AANTAL DEELNEMERS ONLINE: **1**

voortgang module 5 100%

Module 5: Laatste voorbereidingen

neem iemand mee naar het gesprek

Tip 2. Neem iemand mee naar het gesprek.

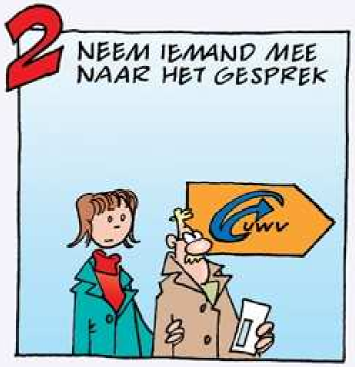

0 | 50 | 100

« vorige | volgende »

MODULES / OPDRACHTEN
FORUM
MIJN WIA

WIA GESPREK .NL

AANTAL DEELNEMERS WIAGESPREK.NL: **178**
AANTAL DEELNEMERS ONLINE: **1**

Institute for Health and Care Research

HOME | COLOFON | PRIVACY | HELP | DISCLAIMER | UITLOGGEN

DE WIA | DE WIA BEOORDELING | NA DE BEOORDELING | ZELFREGIE | LINKS

Wilt u uw medisch CV liever niet op de site invullen? Download hier een blanco Medisch CV.

Medisch CV

dhr. Janssen

Mijn ziekte | Medicijngebruik | Artsen | Ext. factoren | Ziekte en werk | Overzicht

Stap 2 van het Medisch CV is het opschrijven welke medicijnen u gebruikt. Uw medicijnen moet u straks ook meenemen naar het gesprek met de verzekeringsarts. Schrijf overigens alleen de medicijnen op die als laatste door uw behandelaar zijn voorgeschreven en die direct met uw ziekte te maken hebben. Schrijf bij elk medicijn op wat het medicijn precies doet ('werking'), hoe vaak u dit medicijn neemt ('dosis') en hoelang u dit medicijn gebruikt. Druk weer op 'Volgende' om verder te gaan

Uw Medicijnen

medicijn	werking	dosis	gebruik sinds
ibuprofen	pijnstiller	3x per dag	2 jaar

volgende »

medicijn toevoegen

MODULES / OPDRACHTEN

FORUM

MIJN WIA

MEDISCH CV

KALENDER / DAGBOEK

WIA CHECKLIST

WIA METER

MIJN PROFIEL

WIA GESPREK .NL

AANTAL DEELNEMERS WIAGESPREK.NL: **178**

AANTAL DEELNEMERS ONLINE: **1**
