# Supplementary material for: Empowerment of disability benefit claimants through an interactive website: design of a randomized controlled trial
Source: BMC Med Inform Decis Mak. 2009 May 10;9:23. doi: 10.1186/1472-6947-9-23 (PMC2689177; doi:10.1186/1472-6947-9-23)
Supplement: Additional file 2 — Questionnaires. Detailed description of the questionnaires used in this study. [file 1472-6947-9-23-S2.pdf]

Institute for Health and Care Research

Questionnaires

primary outcome

Empowerment
The VrijBaan questionnaire is developed by Tessa Zaeyen and Jan de Koning.
Currently, they are working on an article that describes the development of this instrument.
For more information, please contact Tessa Zaeyen, tzaeyen@pluryn.nl; or Jan de Koning, info@dekoningadvies.nl

secondary outcomes

Claimants satisfaction
Developed by AStri, for use please contact Joke Harmsen, joke.harmsen@uwv.nl

Het gesprek met de verzekeringsarts van UWV

WIA GESPREK .nl

Deze vragenlijst gaat over het gesprek dat u heeft gehad met de verzekeringsarts.

3. Als u uw tevredenheid over uw gesprek bij de verzekeringsarts zou uitdrukken in een rapportcijfer, welk cijfer zou u dan geven?

	1	2	3	4	5	6	7	8	9	10
cijfer:	○	○	○	○	○	○	○	○	○	○

4. Geef aan in hoeverre u het eens bent met de volgende uitspraken over het gesprek bij de verzekeringsarts:
*Bij een vraag met een * kunt u "niet van toepassing" (nvt) invullen.*

	helemaal mee oneens	*mee oneens*	*niet mee eens/niet mee oneens*	*mee eens*	*helemaal mee eens*	nvt	
de verzekeringsarts nam de tijd voor mijn verhaal.	○	○	○	○	○	○	
de verzekeringsarts ging goed in op de dingen die ik vertelde.	○	○	○	○	○	○	
de verzekeringsarts reageerde goed op waar ik aangaf de meeste klachten te hebben.	○	○	○	○	○	○	
de verzekeringsarts nam mijn klachten en beperkingen serieus.	○	○	○	○	○	○	
de verzekeringsarts vroeg hoe ik me voelde.	○	○	○	○	○	○	
de verzekeringsarts stelde me aan het begin van het gesprek op mijn gemak.	○	○	○	○	○	○	
de verzekeringsarts gaf me de gelegenheid om vragen te stellen.	○	○	○	○	○	○	
de verzekeringsarts kon goed met mijn emoties omgaan.	○	○	○	○	○	○	*

5. Geef aan in hoeverre u het eens bent met de volgende uitspraken:

*Bij een vraag met een * kunt u "niet van toepassing" (nvt) invullen.*

	helemaal mee oneens	mee oneens	niet mee eens/niet mee oneens	mee eens	helemaal mee eens	nvt	
de verzekeringsarts maakte kwetsende opmerkingen.	○	○	○	○	○	○	
de verzekeringsarts stelde suggestieve vragen.	○	○	○	○	○	○	
de verzekeringsarts behandelde mij alsof ik misbruik wilde maken van de regeling.	○	○	○	○	○	○	
de verzekeringsarts ging respectvol met me om.	○	○	○	○	○	○	
de verzekeringsarts zat de hele tijd in het dossier of op zijn beeldscherm te kijken.	○	○	○	○	○	○	
het was me duidelijk wat me in de beoordeling te wachten stond.	○	○	○	○	○	○	
het was me duidelijk dat de verzekeringsarts onderzoekt wat ik nog wel kan met mijn klachten.	○	○	○	○	○	○	
de verzekeringsarts legde steeds uit waarom hij/zij dingen deed of naliet tijdens de beoordeling.	○	○	○	○	○	○	
de verzekeringsarts legde duidelijk uit hoe hij/zij tot het oordeel over mijn werkmogelijkheden is gekomen.	○	○	○	○	○	○	
de verzekeringsarts gaf voldoende uitleg over wat er na dit gesprek ging gebeuren.	○	○	○	○	○	○	
de strekking van het gesprek kwam overeen met het verslag dat ik later ontving.	○	○	○	○	○	○	*

Druk op "verder" om door te gaan.

Het gesprek met de verzekeringsarts van UWV

Deze vragenlijst gaat over het gesprek dat u heeft gehad met de verzekeringsarts.

6. Geef aan in hoeverre u het eens bent met de volgende uitspraken:

*Bij een vraag met een * kunt u "niet van toepassing" (nvt) invullen.*

	helemaal mee oneens	mee oneens	niet mee eens/niet mee oneens	mee eens	helemaal mee eens	nvt	
de verzekeringsarts besteedde in het lichamelijk onderzoek voldoende aandacht aan mijn beperkingen.	○	○	○	○	○	○	*
de verzekeringsarts raadpleegde (gegevens van) mijn arts/behandelaar.	○	○	○	○	○	○	*
de verzekeringsarts besteedde voldoende aandacht aan mijn medisch CV.	○	○	○	○	○	○	*
de verzekeringsarts hield in zijn/haar oordeel voldoende rekening met mijn klachten en beperkingen.	○	○	○	○	○	○	
ik had de indruk dat de verzekeringsarts de uitslag vooraf al had bepaald.	○	○	○	○	○	○	
de verzekeringsarts had voldoende kennis over de beperkingen die mijn aandoening met zich meebrengt voor werken.	○	○	○	○	○	○	
de verzekeringsarts stelde goede vragen over wat ik allemaal wel en niet kan.	○	○	○	○	○	○	
de verzekeringsarts gaf de indruk dat hij/zij de bevindingen van mijn artsen/behandelaren begreep.	○	○	○	○	○	○	
de verzekeringsarts onderzocht mij op een manier zoals een huisarts of specialist doet.	○	○	○	○	○	○	
de verzekeringsarts gaf me het gevoel dat hij/zij alleen maar de regeltjes aan het afwerken was.	○	○	○	○	○	○	

Physician satisfaction

5. Als u uw tevredenheid over het gesprek met de client zou uitdrukken in een rapportcijfer, welk cijfer zou u dan geven?

1	2	3	4	5	6	7	8	9	10
○	○	○	○	○	○	○	○	○	○

6. Geef bij de volgende stellingen aan in hoeverre u het hiermee eens bent.

	helemaal mee oneens	*mee oneens*	*niet mee eens/niet mee oneens*	*mee eens*	*helemaal mee eens*
Over het algemeen verliep het gesprek met de client prettig.	○	○	○	○	○
Ik had het idee dat de client eerlijk en oprecht was tijdens het gesprek.	○	○	○	○	○
De client kon duidelijk aangeven wat zijn/haar klachten en beperkingen waren.	○	○	○	○	○
Ik heb een goede inschatting kunnen maken van de mogelijkheden en beperkingen van de client.	○	○	○	○	○
Ik had het idee dat de client na afloop van het gesprek goed wist waar hij/zij aan toe was.	○	○	○	○	○
Bij het invullen van de FML heb ik nauwelijks getwijfeld over mijn beslissingen.	○	○	○	○	○
Ik had het idee dat de client zijn klachten overdreef om zo meer kans te maken op een uitkering.	○	○	○	○	○
De client nam tijdens het gesprek een constructieve houding aan.	○	○	○	○	○
Ik heb het gesprek met de client correct kunnen afhandelen.	○	○	○	○	○
De client had zich goed voorbereid op het gesprek.	○	○	○	○	○
De client was erg mondig.	○	○	○	○	○

7. In hoeverre had de client een actieve rol tijdens het gesprek?

	1	2	3	4	5	
de client had een erg <u>passieve</u> rol	○	○	○	○	○	de client had een erg <u>actieve</u> rol

8. Hoeveel tijd was u kwijt aan het gesprek met de client (in minuten)?

Institute for Health and Care Research

Claimants perceived justice

3. Ik vind de uitslag van de WIA–beoordeling eerlijk...

Indien de vraag niet van toepassing is kunt u "neutraal" invullen.

	Helemaal niet			***Neutraal***			***Helemaal wel***
... in verhouding tot andere mensen.	☐	☐	☐	☐	☐	☐	☐
... met het oog op datgene waar ik recht op heb.	☐	☐	☐	☐	☐	☐	☐
... als ik bedenk wat ik behoor te krijgen volgens de regelgeving.	☐	☐	☐	☐	☐	☐	☐
... gezien mijn inzet in de maatschappij.	☐	☐	☐	☐	☐	☐	☐
... wat betreft mijn positie in de samenleving.	☐	☐	☐	☐	☐	☐	☐
... gezien eerdere ervaringen met UWV.	☐	☐	☐	☐	☐	☐	☐
... ten opzichte van problemen die ik al heb meegemaakt met de overheid.	☐	☐	☐	☐	☐	☐	☐

4. Ik denk dat UWV...

Indien de vraag niet van toepassing is kunt u "neutraal" invullen.

	Helemaal niet			***Neutraal***			***Helemaal wel***
... mij op dezelfde manier heeft behandeld als anderen.	☐	☐	☐	☐	☐	☐	☐
... dezelfde procedures heeft gevolgd als bij anderen.	☐	☐	☐	☐	☐	☐	☐
... zich goed heeft laten informeren.	☐	☐	☐	☐	☐	☐	☐
... alle belangrijke informatie heeft gehad.	☐	☐	☐	☐	☐	☐	☐
... alle nodige informatie heeft gebruikt.	☐	☐	☐	☐	☐	☐	☐
... belangrijke personen en instanties hebben betrokken.	☐	☐	☐	☐	☐	☐	☐
... een goede afweging heeft gemaakt tussen mijn belang en hun eigen belang.	☐	☐	☐	☐	☐	☐	☐
... op een goede manier is omgegaan als ik vragen had of als iets niet duidelijk was.	☐	☐	☐	☐	☐	☐	☐
... mij de kans heeft gegeven om mogelijke bezwaren te laten horen.	☐	☐	☐	☐	☐	☐	☐
... op een goede manier is omgegaan met mogelijke bezwaren.	☐	☐	☐	☐	☐	☐	☐
... op een eerlijke manier is omgegaan met mijn klachten.	☐	☐	☐	☐	☐	☐	☐
... rekening heeft gehouden met verschillende meningen van mensen.	☐	☐	☐	☐	☐	☐	☐

Coping strategy

7. Klik per uitspraak het antwoord aan dat het meest van toepassing is voor uw voorbereiding op het gesprek met de arts van UWV:

	geheel niet	*een beetje*	*tamelijk veel*	*zeer veel*
Ik concentreer mij op wat ik moet doen of gaan vertellen				
Ik probeer beter te begrijpen waarom het gesprek zal plaatsvinden				
Ik doe dingen om mijn gedachten af te leiden				
Ik heb het gevoel dat de tijd het wel zal leren – ik hoef alleen af te wachten				
Ik ga door alsof er geen gesprek zal zijn				
Ik probeer op een creatieve manier me voor te bereiden op het gesprek				
Ik probeer het gesprek te vergeten				
Ik wacht af wat er zal gebeuren				
Ik maak een plan ter voorbereiding				
Ik probeer het even van me af te zetten door bijvoorbeeld rust te nemen				
Ik probeer rustig te overwegen hoe ik het gesprek zal aanpakken				
Ik ga voet bij stuk houden en vechten voor wat ik wil bereiken tijdens het gesprek				
Ik weet wat er gedaan moet worden, dus ik span mij extra in om het voor elkaar te krijgen				
Ik bedenk een aantal verschillende strategieen voor het gesprek				
Ik accepteer dat het gesprek toch door zal gaan				

8. Klik per uitspraak het antwoord aan dat het meest van toepassing is voor uw voorbereiding op het gesprek met de arts van UWV:

	geheel niet	*een beetje*	*tamelijk veel*	*zeer veel*
Ik praat met iemand om meer over het gesprek te weten te komen				
Ik probeer mijn gevoelens voor mezelf te houden				
Ik aanvaard medeleven en begrip van iemand				
Ik uit mijn gevoelens over het gesprek op de een of andere manier				
Ik vraag advies aan een partner, familielid of vriend(in)				
Ik praat met iemand over hoe ik me voel				
Ik ga in gedachten na wat ik zal zeggen of doen				
Ik probeer de dingen vanuit het standpunt van de arts te bekijken				
